# Effect of the total flavonoids of *Dracocephalum moldavica L.* on metabolic associated fatty liver disease in rats 

**DOI:** 10.3389/fphar.2025.1549515

**Published:** 2025-05-15

**Authors:** Xiaoyu Sun, Na Ge, Qingqing Liang, Qian Wang, Hui Yu, Min Jin

**Affiliations:** ^1^ The School of Public Health, Baotou Medical College, Baotou, China; ^2^ The School of Basic Medicine and Forensic Sciences, Baotou Medical College, Baotou, China

**Keywords:** MAFLD, intestinal mucosal barrier, TLR4/MyD88/NF-κB, liver impairment, gut microbiota

## Abstract

**Introduction:**

Metabolic-associated fatty liver disease (MAFLD) is a liver disease syndrome. The total flavonoids of Dracocephalum moldavica L. (D. moldavica), the main active components of D. moldavica, have been demonstrated not only to have anti-inflammatory and antioxidant effects but also to regulate gut microbiota. However, the mechanism by which it improves MAFLD is unclear. So we want to investigate how the total flavonoids of D. moldavica alleviate high-fat diet (HFD)-induced MAFLD in rats.

**Methods:**

Firstly, MAFLD rat models were established by feeding with HFD, while the total flavonoids of D. moldavica were administered via gavage. Then, the experiments analyzed the changes of gut microbiota by the 16S rRNA sequencing and detected intestinal barrier permeability and liver inflammation to explore the mechanism of the total flavonoids of D. moldavica in the prevention and treatment of MAFLD.

**Results:**

We found that the total flavonoids of D. moldavica reduced systemic inflammation and could alleviate serum and liver lipid metabolism disorders in MAFLD rats. The results of the 16S rRNA sequencing demonstrated that the total flavonoids of D. moldavica increased the abundance and diversity of gut microbiota. Furthermore, the total flavonoids of D. moldavica have been demonstrated to enhance the intestinal mucosal barrier function, reduce LPS translocation, and inhibit the activation of hepatic TLR4/MyD88/NF-κB pathway. This could effectively ameliorate the hepatic lesions in MAFLD rats.

**Conclusions:**

The aforementioned outcomes indicate that the total flavonoids ofD moldavica may potentially alleviate MAFLD by modulating gut microbiota, intestinal mucosal barrier and hepatic inflammation.

## 1 Introduction

Metabolic associated fatty liver disease (MAFLD, formerly known as non-alcoholic fatty liver disease, NAFLD) is a complex metabolic disorder characterised by oxidative stress, fat accumulation and liver inflammation (Younossi et al., 2016). MAFLD is a progressive disease that encompasses a spectrum of pathologies, including hepatic steatosis, inflammation, fibrosis, cirrhosis, liver failure, and even hepatocellular carcinoma (Targher et al., 2024). In recent years, the prevalence of MAFLD has been on the rise, with a global prevalence rate of up to 25%. This poses a significant burden on human health and socio-economics, and it is a critical medical problem that needs to be addressed with urgency (Duell et al., 2022).

The pathogenesis of MAFLD remains uncertain, with the “two hit” mechanism regarded as the classic hypothesis. Insulin resistance in the body is postulated to result in lipid accumulation in hepatocytes, which subsequently leads to a reduction in the anti-inflammatory function of the liver. The “first-hit” refers to liver oxygenation and blood supply, while the “second-strike” is the accumulation of hepatocyte lipids, which further damages hepatocytes due to the action of inflammatory factors, endoplasmic reticulum stress and oxidative stress. The “second strike” refers to the accumulation of hepatocyte lipids, further damage to hepatocytes under the action of inflammatory factors, endoplasmic reticulum stress and oxidative stress, which ultimately results in inflammation of liver tissues, and the progression of simple cellular steatosis to MAFLD (Maher et al., 2008). In recent years, with the deepening of the research, the “multiple-strike theory” has gradually replaced the “second-strike theory”. This theory proposes that the gut microbiota can also regulate a number of processes, including the inflammatory response, insulin resistance, bile acid and choline metabolism (Buzzetti et al., 2016).

The gut microbiota plays an important role in maintaining host health, including the digestion of dietary fibre, synthesis of nutrients and vitamins, defense against pathogens, and regulation of the immune system (Zhang L. et al., 2023; Dai et al., 2023a; Liu P. et al., 2021). In recent years, it has been demonstrated that liver disease and gut microbiota dysbiosis are closely related (Luo et al., 2023). It has been demonstrated that dysregulation of the gut microbiota and its metabolites may contribute to the development of MAFLD through a variety of mechanisms, including disruption of fat metabolism in the liver, promotion of fat accumulation and lipotoxicity (Xia et al., 2023; Park et al., 2021; Liu et al., 2022). In addition, dysbiosis of the gut microbiota can lead to intestinal barrier dysfunction.

Ma et al. (2013) initially proposed that human MAFLD is associated with increased intestinal permeability. For the past few years, a large amount of literature has indicated that MAFLD/NASH patients frequently exhibit varying degrees of intestinal barrier dysfunction (Maresca et al., 2024). Intestinal barrier dysfunction results in overgrowth of bacteria in the intestinal lumen, disruption of the microecology and produce a large number of toxic metabolites and endotoxins. The endotoxins (LPS) that enter the blood circulation have the potential to directly injure the epithelial cells of the intestinal mucosa, resulting in contraction of the intestinal microvessel and ischemia and hypoxia of the intestinal tissues. This, in turn, can lead to the production of a large number of reactive oxygen species (ROS), which can further exacerbate the damage to the intestinal barrier. Conversely, blood-entered LPS also acts on the liver and initiates an inflammatory response with hepatic Kupffer cells and intracellular Toll-like receptor (TLR), in which the LPS/TLR4/MyD88/NF-kB pathway, which has been shown to play a key role in MAFLD (Ren et al., 2024a; Suk and Kim, 2019; Liu X. et al., 2021; Shen et al., 2022; Li et al., 2023). An essential role in this process is played by the intestinal mucosal barrier, a natural barrier between the gut microbiota and the intestinal lumen. This selectively controls the flow of useful substances such as nutrients, ions, and water into and out of the lumen. It also prevents the entry of harmful substances such as the endotoxin LPS, including bacteria and their metabolites (Koh et al., 2016).

At present, there is no approved therapeutic drug for MAFLD. The primary treatment modality is through weight reduction and lifestyle intervention. The extensive attention afforded to natural products is a consequence of their advantages, including the existence of multiple pathways and multiple targets, as well as their high safety profile. Flavonoids extracted by ethanol reflux from *Dracocephalum moldavica L.* have attracted the attention of many researchers in recent years because of their wide range of effects. The total flavonoids of *Dracocephalum moldavica L.* mainly have been demonstrated to possess a range of biological activities, including anti-inflammatory, antioxidant and alleviating insulin resistance (Nie et al., 2021; Aćimović et al., 2022). Previously, our research group also demonstrated that the total flavonoids of *Dracocephalum moldavica L.* can exert their anti-colitis effects through regulating the gut microbiota and TLR4/NF-ΚB pathway (Gang et al., 2023). Based on the favourable anti-inflammatory and gut microbiota regulation effects of the total flavonoids of *Dracocephalum moldavica* L., we will investigate the protective effects of the total flavonoids of *Dracocephalum moldavica L.* against MAFLD from the perspectives of inflammation, lipid metabolism and intestinal barrier.

In this study, we used HFD to establish rat MAFLD models and used the total flavonoids of *Dracocephalum moldavica L.* to intervene in MAFLD rats. The objective of this study was to investigate the preliminary intervention effect of the total flavonoids of *Dracocephalum moldavica L.* on MAFLD. To this end, serum inflammation, lipid metabolism, and liver pathology sections were tested in rats. Subsequently, the protective effect of the total flavonoids of *Dracocephalum moldavica L.* on the intestinal barrier of MAFLD rats was further explored by 16S rRNA gut microbiota sequencing, ileal pathology sections, RT-qPCR, Western blotting, and immunohistochemistry.

## 2 Experimental procedures

### 2.1 Extraction and component identification of total flavonoids from *Dracocephalum* m*oldavica L*.

*Dracocephalum moldavica L.* was cultivated in the medicinal plant garden of Baotou Medical College. After drying, the herbs were ground into powder. A total of 150 g of *Dracocephalum moldavica L.* powder was weighed and placed in a three-necked flask. Using a graduated cylinder, 900 mL of absolute ethanol and 600 mL of deionized water were measured and mixed to a total volume of 1,500 mL, resulting in a 60% ethanol solution. The solution was poured into the three-necked flask, stirred, and allowed to soak for 12 h. The mixture was then heated under reflux at 60°C in a water bath for 2 h. After vacuum filtration, the extracted liquid was obtained and freeze-dried to a powder. Once the total flavonoid concentration was confirmed to be 80% by comparison with standard samples, the components of the total flavonoids from *Dracocephalum moldavica L*. were identified using the HPLC method.

### 2.2 Establishment of MAFLD rat models

The study was approved by the Animal Ethics Review Committee of Baotou Medical College. Every effort was made to minimize the pain and discomfort of the animals. Animal experiments were performed at the Animal Center of Baotou Medical College (Ethical Review Approval for Animals, Baotou Medical College [2023] No.6).

Forty 5-week-old SD rats with body mass of 150 g–160 g were purchased from Beijing, China. A 12-h circadian rhythm was maintained indoors at a temperature of 25°C ± 1C and a relative humidity of 55% ± 5% for 1 week of acclimatization rearing. During the acclimatization rearing period, normal feed was fed. Then, they were randomly divided into four groups according to body weight: low-fat feed control group (C Group), high-fat feed model group (M Group), *Dracocephalum moldavica L.* total flavonoid high-dose group (DH Group), andtotal flavonoid low-dose group (DL Group), with 10 animals in each group.

To construct the MAFLD model, M group, DH and DL were given high-fat diet (D12450,Xiao Shu You Tai Biotechnology Co.,Ltd.) and C group was given low-fat diet (D12450H, Xiao Shu You Tai Biotechnology Co.,Ltd.) and fed for 12 weeks. During this period, the DH group was gavaged with 200 mg/kg of total flavonoids of *Dracocephalum moldavica L.* per day, the DL group was gavaged with 100 mg/kg of total flavonoids of *Dracocephalum moldavica L.* per day (the dose of total flavonoids of *Dracocephalum moldavica L.* per day was determined by pre-testing), and equal amounts of saline were given to the C and M groups per day by gavage. The rats were weighed once a week during the experiment. At the end of the 12th week, the rats in each group were fasted for 12 h, allowed water, and anesthetized with 2% pentobarbital sodium. After complete anesthesia, blood was collected from the abdominal aorta, and the blood was allowed to stand at room temperature for 1 h. The blood was centrifuged in a low-temperature centrifuge at 4°C for 10 min at 3,000 rpm, and the upper layer of the blood serum was separated and stored at −80°C. The liver tissues and liver tissues were collected from the rats in each group. The liver and ileum tissues were collected from each group of rats, and part of the tissues were placed in 4% paraformaldehyde fixative for histopathological examination, and part of the tissues were quick-frozen in liquid nitrogen and then placed at −80°C for RT-qPCR or Western blotting experiments.

### 2.3 Serum biochemical analysis

AST, ALT, TC, TG, HDL-C and LDL-C levels were determined by using 2.1 treated serum and following the steps of Beijing Solepol Assay Kit instructions.

### 2.4 Liver biochemical tests

Weigh 100 mg of liver tissue, add the extraction solution according to the instructions of Beijing Solepol Assay Kit, homogenize in ice bath, 10000 g, centrifuge at 4°C for 10 min, take the supernatant, and then measure TC, TG, HDL-C, LDL-C, which are related to the lipid metabolism of rat liver according to the instructions.

### 2.5 ELISA

Rat systemic inflammatory factors TNF-α, IL-6 and IL-1β, and LPS levels were measured using ELISA kits (Quanzhou Jiubang Biotechnology Co., Ltd.) using 2. 1-treated serum.

### 2.6 Tissue staining

2.1 Fixed tissue samples, dehydrated paraffin-embedded, were cut into 3um thick paraffin sections, and finally the sections were stained with hematoxylin and eosin. Liver tissues were frozen and OCT-embedded and sectioned, and stained with oil red O and hematoxylin. All sections were photographed under the OLYMUPS BX41 system.

### 2.7 Western blotting

100 mg of liver tissue or ileum tissue was cut and added with 1000 μL lysate and 10 μL PMSF, homogenized, centrifuged at 12000 rpm for 10 min at 4°C, and the supernatant was taken to determine the concentration of the sample proteins according to the instructions of the BCA assay kit. Then, the proteins were separated by SDS-PAGE and transferred into a PVDF membrane, which was closed with a closure solution. After adding primary antibody, the membrane was incubated overnight at 4°C. After washing the membrane, secondary antibody was added and incubated for 60 min, the membrane was washed, luminescent solution was added and the film was developed. The information of antibodies is shown in Table 1. The film was scanned by a scanner, and the grayscale value was analyzed by ImageJ software.

**TABLE 1:** The information of Antibody.

Antibody name	Manufacturer	Catalog number	Dilution factor	Molecular weigh
ZO-1	Affinity	AF5145	1:1100	195 kDa
Occludin	Affinity	DF7504	1:1000	59 kDa
TLR4	Affinity	AF7017	1:1200	120 kDa
MYD88	Affinity	AF5195	1:800	33 kDa
NFKB P65	ABCAM	AB16502	1:1000	65 kDa
P-NFKB P65	ABCAM	AB76302	1:1000	65 kDa
GAPDH	Affinity	AF7021	1:10000	37 kDa

### 2.8 Immunohistochemistry

Ileal tissue sections were deparaffinized for antigen repair. after BSA closure, primary antibody was added and incubated overnight at 4°C. After rinsing with PBS, secondary antibody was added and incubated for 20 min at 37°C. After rinsing with PBS, DAB was used to develop the color, and hematoxylin was used for re-staining after washing with water.

### 2.9 Quantitative real-time PCR

Liver tissue total RNA was extracted according to the AxyPrep total RNA small volume preparation kit (Axygen Co., Ltd.). Total RNA wasconverted to cDNA using a reverse transcription kit (Nanjing Novozymes Biotechnology Co., Ltd.). cDNA was obtained from reverse transcription according to the instructi ons of the qPCR kit (Nanjing Novozymes Biotechnology Co., Ltd.) and then a nalysed on a LightCycle 96 machine. The expression level data were normalize d to the expression level of GAPDH, and the 2^−ΔΔCT^ method was used for cal culating the expression. The primer sequences are listed in Table 2.

**TABLE 2:** Primer sequences.

Gene	Forward sequence	Reverse sequence
TLR4	CAG​AAT​GAG​GAC​TGG​GTG​AGA​AAC​G	TCC​TGG​ATG​ATG​TTG​GCA​GCA​ATG
MyD88	GTC​TCC​AGG​TGT​CCA​ACA​GAA​GC	GTC​GCA​GAT​AGT​GAT​GAA​CCG​TAG​G
NF-κB	TGT​GGT​GGA​GGA​CTT​GCT​GAG​G	AGT​GCT​GCC​TTG​CTG​TTC​TTG​AG
GAPDH	ACG​GCA​AGT​TCA​ACG​GCA​CAG	CGA​CAT​ACT​CAG​CAC​CAG​CAT​CAC

### 2.10 Long read 16S rRNA gene sequencing

16S rRNA amplicon sequencing was conducted on a MinION nanopore sequencer (Oxford Nanopore Technologies, Oxford, United Kingdom). The amplicon library was prepared using the 16S Barcoding Kit 1-24 (SQK-16S024, Oxford Nanopore Technologies, Oxford, United Kingdom).For the polymerase chain reaction (PCR) amplification and barcoding, 15 ng of template DNA extracted from fecal samples were added to the Long Amp Hot Start Taq 2X Master Mix (New England Biolabs, Ipswich, MA, United States). The initial denaturation was conducted at 95°C, followed by 35 cycles of 20 s at 95°C, 30 s at 55°C, 2 min at 65°C, and a final extension step of 5 min at 65°C. Purification of the barcoded amplicons was performed using the AMPure XP Beads (Beckman Coulter, Brea, CA, United States) in accordance with the instructions provided by Nanopore. Samples were then quantified using a Qubit fluorometer (Life Technologies, Carlsbad, CA, United States) and pooled in an equimolar ratio to a total of 50 ng–100 ng in 10 µL. Subsequently, the pooled library was loaded into a R9.4.1 flow cell and run in accordance with the manufacturer’s instructions. MINKNOW software version 19.12.5 was employed for data acquisition.

### 2.11 Long-read 16S bioinformatic analysis

The raw sequences were base called with Guppy v4.4.2, utilising the trim barcodes’ function and quality score (qscore) filtering, with a minimum score of 7. The microbial community profiles for each sample were generated with Emu v1.0.2, utilising the default parameters and database. Alpha and beta diversity analyses for microbiome samples were conducted in Python 3.8 using the scikit-bio v0.4.1 diversity package. Alpha diversity values were calculated for each sample using the Shannon and Simpson indices, while beta diversity was determined using the Weighted UniFrac metric. A phylogenetic tree was generated from the Emu default database using the taxdump to tree.py Python script in Biocore, with each branch length set to 1. A principal coordinate analysis (PCoA) distance matrix was then generated, and statistical differences between the two groups were calculated with 9,999 simulated ANOSIM permutations. To facilitate visualisation of these values, a Matplotlib v.3.3.3 beta diversity scatter plot was generated, with 95% confidence ellipses drawn around each group. This was achieved through the use of Matplotlib two-dimensional confidence ellipse source code. To illustrate the average relative abundance of bacteria in each group, stacked bar plots were generated with GraphPad Prism version 9. The log2 fold change was calculated between two samples from the same participant by taking the log2 of the percent relative abundance of the later time point divided by an earlier time point.In order to prevent the introduction of mathematical errors, values representing zero relative abundance were transformed to the value of one ten-billionth.

### 2.12 Data analysis

Data are expressed as mean ± standard deviation. Statistical analysis was performed using SPSS 22.0 and GraphPad Prism 8.0 for graph design. Comparisons between groups were made using one-way ANOVA and multiple comparisons, with *P* < 0.05 indicating a significant difference.

## 3 Results

The experiment employed mass spectrometry to analyze the main chemical components of the total flavonoids of *Dracocephalum moldavica L.*, with results presented in Supplementary Material S1suppp. It was found that the total flavonoids of *Dracocephalum moldavica L.* are primarily composed of flavonoid components such as quercetin, luteolin, isorhamnetin, and apigenin.

### 3.1 The effects of the total flavonoids of *Dracocephalum moldavica L.* on body weight and liver of rats in various groups were investigated

**FIGURE 1:**
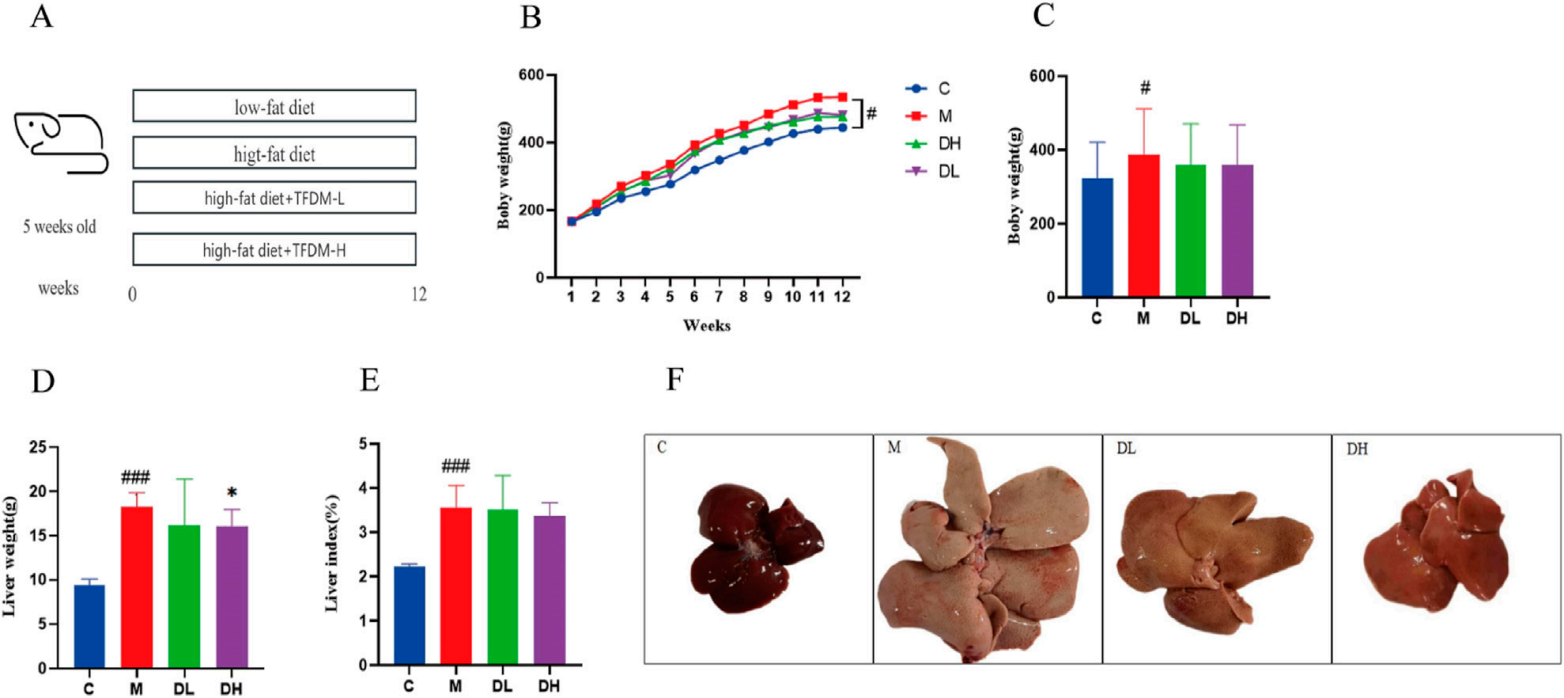
The effects of the total flavonoids of *Dracocephalum moldavica L*. on body weight and liver of rats in various groups. **(A)** Experimental flow chart. **(B)** Temporal changes in body weight of each group (n = 8). **(C)** Body weight (n = 8). **(D)** Liver weight (n = 8). **(E)** Liver index (n = 8). **(F)** Overall liver morphology. Data are shown as mean values ± *S*. Compared with the C group, ^#^ *P* < 0.05,^###^ *P* < 0.001, and compared with the M group,^*^ *P* < 0.05.

Given the close association between MAFLD and obesity, the experiments recorded the body weight, liver weight and liver index (expressed as the ratio of liver weight to body weight) of the rats. Figure 1A shows the experimental flow chart. As shown in Figure 1B, following 12 weeks of feeding, the body weights of rats in all groups showed an increasing trend. Among these, the M group demonstrated the most rapid body weight gain, which was significantly different (*P* < 0.05) compared with the C group. Among these, the M group demonstrated the most rapid body weight gain, which was significantly different (P < 0.05) compared with the C group (Figure 1C). The groups DL and DH exhibited a slower rate of body weight growth compared with the M group. The body weight of rats in the groups DL and DH increased slowly compared with those in the M group. Furthermore, the liver weight of the DH group was lower than that of M group (*P* < 0.05) after high dose intervention with the total flavonoids of *Dracocephalum moldavica L.* (Figures 1D, E). The appearance of rat livers could be observed (Figure 1F), the livers of rats in the C group were dark red, with a smooth epidermis and a tight envelope. In contrast, the livers of rats in the M group were obviously yellowish, enlarged in size, and oily to the naked eye. The livers of rats in the DH group were dark red in colour, with a tight envelope, and exhibited a morphology was more similar to that of the C group. The results demonstrated that the liver weight of rats in the DH group was lower than that of rats in the M group (*P* < 0.05) (Figure 1D). The findings indicated that the total flavonoids of *Dracocephalum moldavica L.* could alleviate the weight gain and liver fat accumulation induced by HFD.

### 3.2 The effects of the total flavonoids of *Dracocephalum moldavica L.* on the pathologic and biochemical indices of the liver of rats in various groups

**FIGURE 2:**
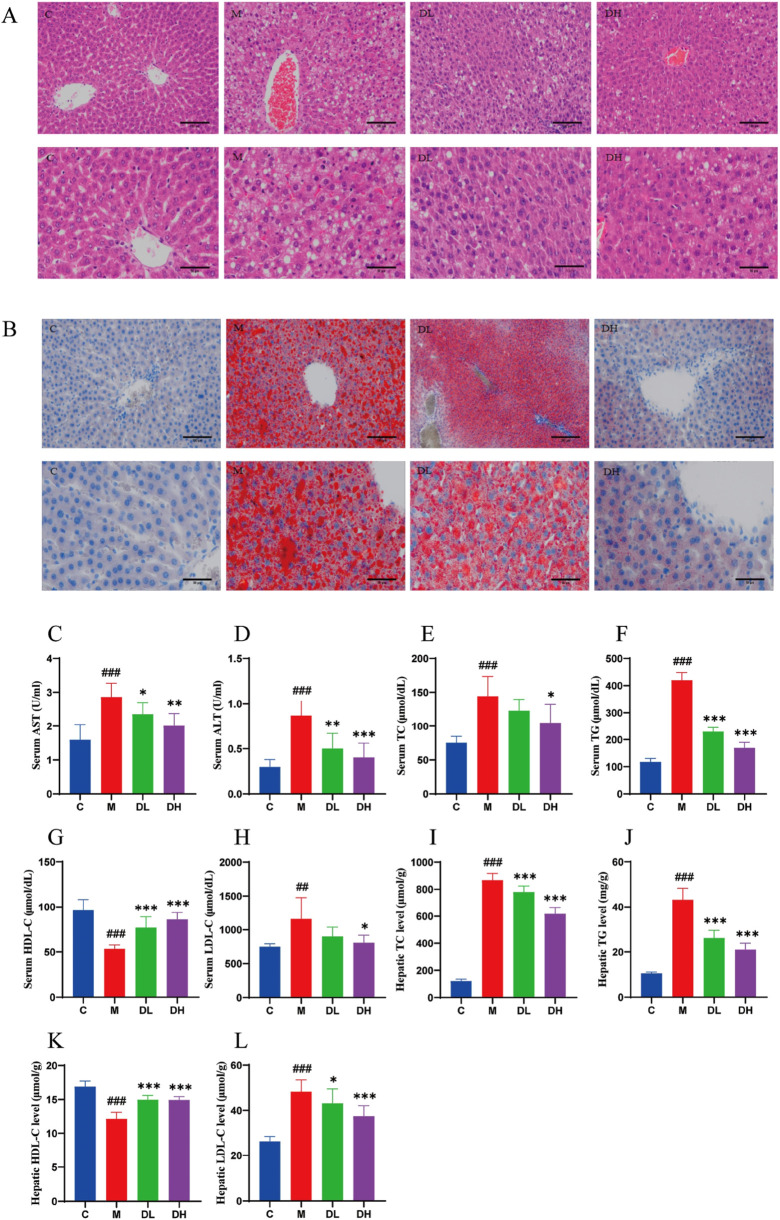
The effects of the total flavonoids of *Dracocephalum moldavica L.* on pathological and biochemical indices of rats liver in various groups. **(A)** HE st aining of liver tissue 200× or 400× (n = 4). **(B)** Liver tissue oil red O stainin g 200× or 400× (n = 4). **(C, D)** Serum AST and ALT levels (n = 8). **(E–L)** Seru m or HepaticTC, TG, HDL-C, LDL-C levels (n = 8). Data are shown as means ± *S*. Compared with the C group,^##^ *P* < 0.01,^###^ *P* < 0.001, and compared with th e M group,^*^ *P* < 0.05,^**^ *P* < 0.01,^***^ *P* < 0.001.

In order to ascertain whether the intervention of the total flavonoids of *Dracocephalum moldavica L.* would could be employed to improve liver injury and lipid accumulation induced by HFD, the liver tissues were stained with HE and Oil Red O. The results of the HE staining indicated (Figure 2A) that the hepatocytes in the livers of rats in the C group were arranged in a regular and tightly packed manner, whereas the liver tissues of the rats in the M group were obviously swollen, and the cytoplasm was filled with lipid droplets of large vacuoles and as well as small vacuoles of varying sizes and quantities. Additionally, inflammatory cell infiltration appeared in a localized area. Following the administration of the total flavonoids of *Dracocephalum moldavica L.*, steatosis was significantly reduced in rats, and sporadic lipid droplets were observed within hepatocytes, and no inflammatory cell infiltration was observed in the DH group. The results of Oil Red O staining showed (Figure 2B) demonstrated the presence of numerous red, tiny lipid droplets of varying sizes with the cytoplasm of rat hepatocytes in the M group. These droplets were observed to fuse with each other, forming larger lipid droplets that occupied the majority or entirety of the cytoplasm. In comparison, the DL and C groups exhibited a reduction in the number of red lipid droplets, with fewer droplets observed in the DL group than in the M group. In the DH group, there were sporadic lipid droplets in the interstitial area of hepatocytes after administration of vanillin. The number of red lipid droplets was significantly reduced in comparison to that of the M group. These results indicated that vanillin administration significantly reduced lipid accumulation in the liver, attenuated hepatic steatosis, and exerted anti-inflammatory effects.

AST and ALT are considered to be commonly used indicators for the diagnosis of liver injury (Han et al., 2019). The levels of AST and ALT were significantly elevated (*P* < 0.001) in the M group (Figures 2C, D), suggesting the presence of liver disease and inflammation. MAFLD initially manifests itself as a lipid accumulation, prompting the conduct of experiments detect the serum and liver levels of triglyceride (TC), total cholesterol (TG), low-density lipoprotein cholesterol (LDL-C) and high-density lipoprotein cholesterol (HDL-C) levels (Figures 2E–L). The results demonstrated that after 12 weeks of HFD feeding, TC, TG, and LDL-C levels were significantly increased (*P* < 0.01 or *P* < 0.001) and HDL-C level was significantly decreased (*P* < 0.001) in the serum and liver of rats in the M group compared with the C group. Whereas, compared with the M group, the DH group significantly reduced TC, TG, and LDL-C in the serum and liver of rats (*P* < 0.05 or *P* < 0.001), while HDL-C was significantly increased (*P* < 0.001). The findings indicated that the total flavonoids of *Dracocephalum moldavica L.* could reduce lipid accumulation and liver injury, and contribute to the improvement of MAFLD.

### 3.3 The effects of the total flavonoids of *Dracocephalum moldavica L.* on the level of inflammation in various groups of rats

**FIGURE 3:**
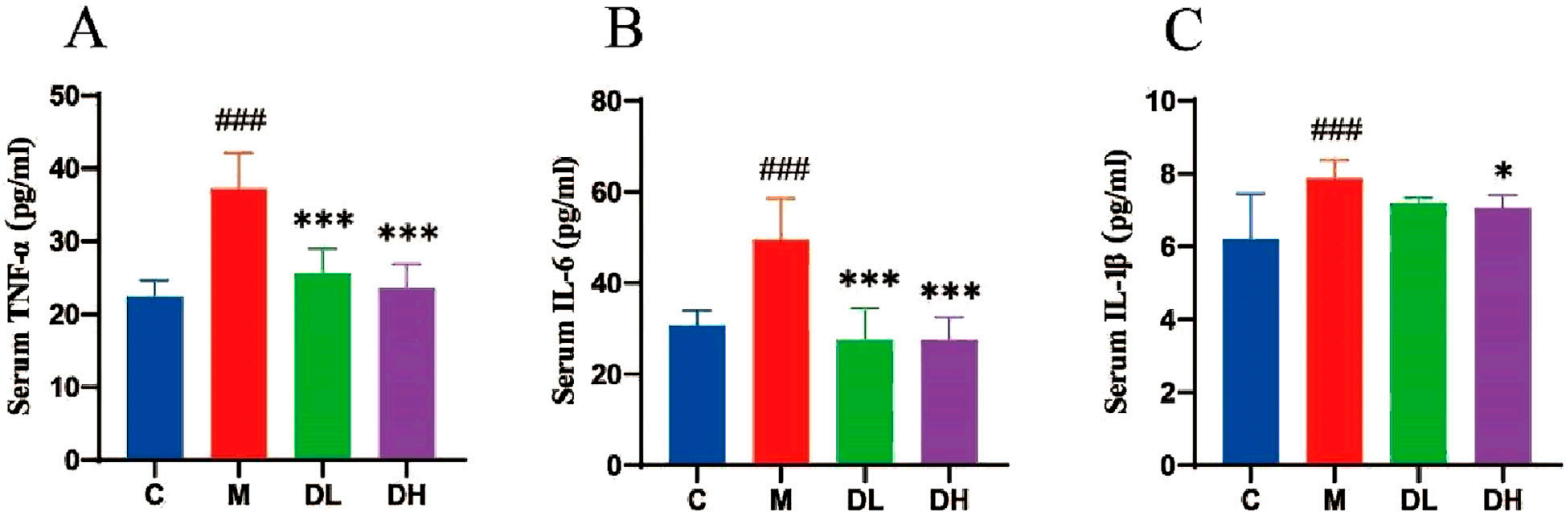
The effects of the total flavonoids of *Dracocephalum moldavica L.* on in flammation levels in rats of all groups. **(A)** Serum TNF-α level (n = 8). **(B)** Ser um IL-6 level (n = 8). **(C)** Serum IL-1β level (n = 8). Data are shown as mean v alues ± *S*. Compared with the C group,^###^ *P* < 0.001, compared with the M gro up,^*^ *P* < 0.05,^***^ *P* < 0.001.

Inflammation is one of the important manifestations of MAFLD, which the experiments sought to address by exploring the potential of total flavonoids of *Dracocephalum moldavica L.* could reduce the level of inflammation in rats. This was assessed by measuring serum levels of tumour necrosis factor-α (TNF-α), interleukin 6 (IL-6), and interleukin 1β (IL-1β) (Figures 3A–C). The results demonstrated that the administration of the total flavonoids of *Dracocephalum moldavica L.* reduced the level of inflammation in rats (*P* < 0.05 or *P* < 0.001).

### 3.4 The effects of the total flavonoids of *Dracocephalum moldavica L.* on the Alpha diversity and beta diversity of gut microbiota of rats in various groups

**FIGURE 4:**
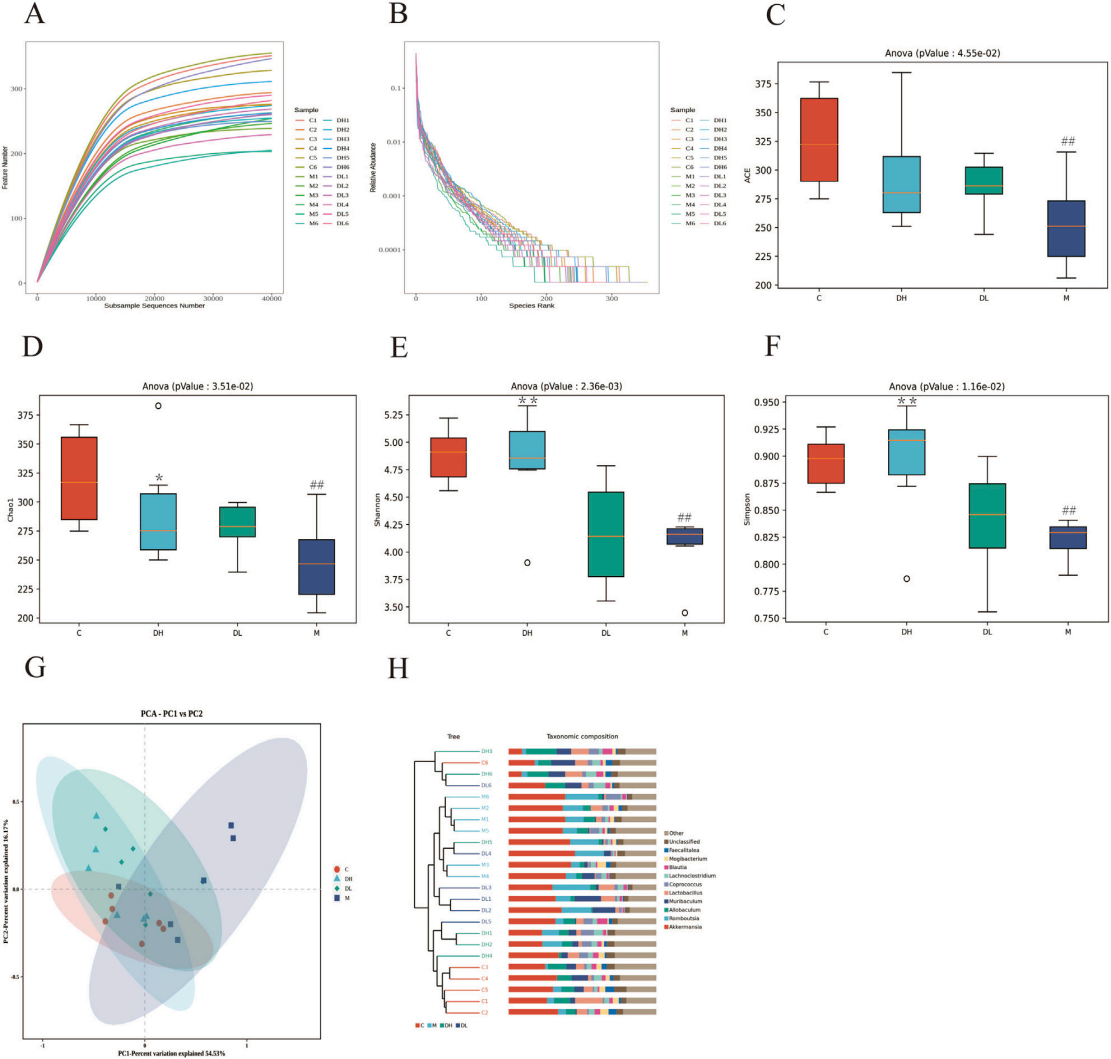
The effects of the total flavonoids of *Dracocephalum moldavica L.* on A lpha diversity and Beta diversity of gut microbiota of rats in various groups. **(A)** Sparsity curves (n = 6). **(B)** Abundance rank curves (n = 6). **(C–F)** ACE, Chao 1, Shannon, and Simpson indices (n = 6). **(G)** PCA analysis (n = 6). **(H)** Cluster t ree analysis (n = 8). Data are shown as mean values ± *S*. Compared with the C group,^##^ *P* < 0.01, compared with the M group,^*^ *P* < 0.05,^**^ *P* < 0.01.

The Rarefaction Curve and Shannon Index Curve indicate that the sequencing volume of the samples is sufficiently adequate and the amount of sequencing data is large enough (Figures 4A, B). In this study, we found that the alpha diversity and abundance of gut microbiota shown by ACE, Chao1, Shannon, and Simpson were reduced in the M group compared to the C group (*P* < 0.01), and the Chao1, Shannon, and Simpson indices were increased in the DH group after the intervention of the total flavonoids of *Dracocephalum moldavica L.* (*P* < 0.05 or *P* < 0.01), indicating that the total flavonoids of *Dracocephalum moldavica L.* could effectively improve the diversity and abundance of the gut microbiota of MAFLD rats (Figures 4C–F). Beta diversity represents the similarity of the species of the microbial samples. PCA plots showed (Figures 4G) that there was a clear separation between the C and M groups, which indicated that the gut microbiota of the two groups differed in their structures and compositions, meanwhile, the DH and DL groups were aggregated with the C group, indicating that the total flavonoids of *Dracocephalum moldavica L.* could restore the structure of the gut microbiota of MAFLD rats. The UPGMA was based on the four distance matrices obtained from the Beta diversity analysis, and the samples were hierarchically clustered by using unweighted pairwise averaging by the R language tool in order to determine the similarity of the species composition between the samples. The sample hierarchical clustering tree of the samples is shown in Figure 4H: the closer the samples were, the shorter the branch lengths were, which indicated that the more similar was the species composition of the two samples, of which. The DH group and the C group showed high species similarity, further indicating that the total flavonoids of *Dracocephalum moldavica L.* could improve gut microbiota disorders in MAFLD rats.

### 3.5 The effects of the total flavonoids of *Dracocephalum moldavica L.* on gut microbiota at the phylum and genus levels

**FIGURE 5:**
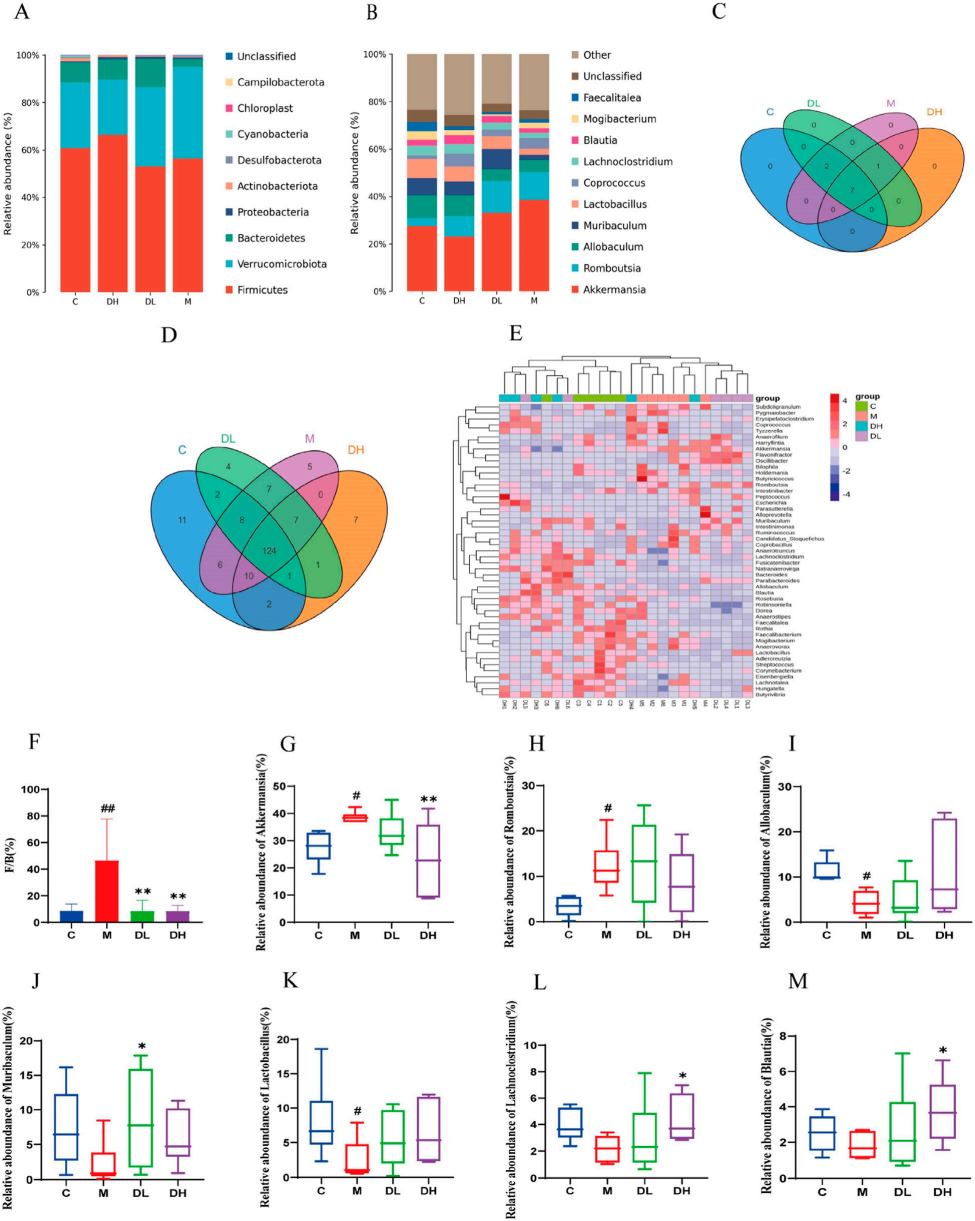
Effect of the total flavonoids of *Dracocephalum moldavica L.* on gut mic robiota at the phylum and genus levels. **(A)** Gate level(n = 6). **(B)** Genus level(n = 6). **(C, D)** Venn diagram at the phylum and genus levels(n = 6). **(E)** Heat map at genus level(n = 6). **(F)** Fimicute and Bacteroidete ratios (n = 6). **(G–M)** Relative abundance of Akkermansia, Romboutsia, Allobaculum, Muribaclum, Lactobacillu s, Lachnoclostridium, Blautia (n = 6). Data are shown as mean values ± *S*. Com pared with the C group,^#^ *P* < 0.05, ^##^ *P* < 0.01, compared with the M group,^*^ *P* < 0.05, ^**^ *P* < 0.01.

To evaluate the effects of the total flavonoids of *Dracocephalum moldavica L.* on the gut microbiota of MAFLD rats, the abundance of the gut microbiota at the phylum and genus levels was analyzed using taxonomy. At the phylum level, the Fimicutes and Bacteroidetes ratios tend to be associated with metabolic diseases such as obesity (Zhang XL. et al., 2023). The Fimicute and Bacteroidete ratios were increased in the M group compared to the C group and decreased after the total flavonoids of *Dracocephalum moldavica L* (Figures 5A, B). The Venn diagram results show that at the genus level, the C, M, DH, and DL groups shared 124 common genera. Specifically, the DH and M groups shared 0 genera, the DH and C groups shared 10 genera, and the DL and C groups shared 2 genera (Figures 5C, D). Differences in the composition of gut microbiota among different groups can also be observed in the Heatmap at the genus level (Figure 5E). The F/B ratio was significantly improved after intervention with the total flavonoids of Dracocephalum moldavica L. (Figure 5F). At the genus level, significant differences were observed in Akkermansia, Romboutsia, Allobaculum, Muribaculum, *Lactobacillus*, Lachnoclostridium, and Blautia. At the genus level, significant differences were observed in Akkermansia, Romboutsia, Allobaculum, Muribaculum, Lactobacillus, Lachnoclostridium, and Blautia (Figures 5G–M). It has been shown that Akkermansia is negatively correlated with intestinal barrier function (Yang et al., 2023). Romboutsia is closely associated with the inflammatory response and the level of Romboutsia was significantly higher in the M group as compared to the C group. The abundance of beneficial bacteria such as Muribaclum, Lachnoclostridium, and Blautia were significantly higher after the total flavonoids of *Dracocephalum moldavica L.* intervention (*P* < 0.05).

### 3.6 The total flavonoids of *Dracocephalum moldavica L.* restore intestinal barrier function in MAFLD rats

**FIGURE 6:**
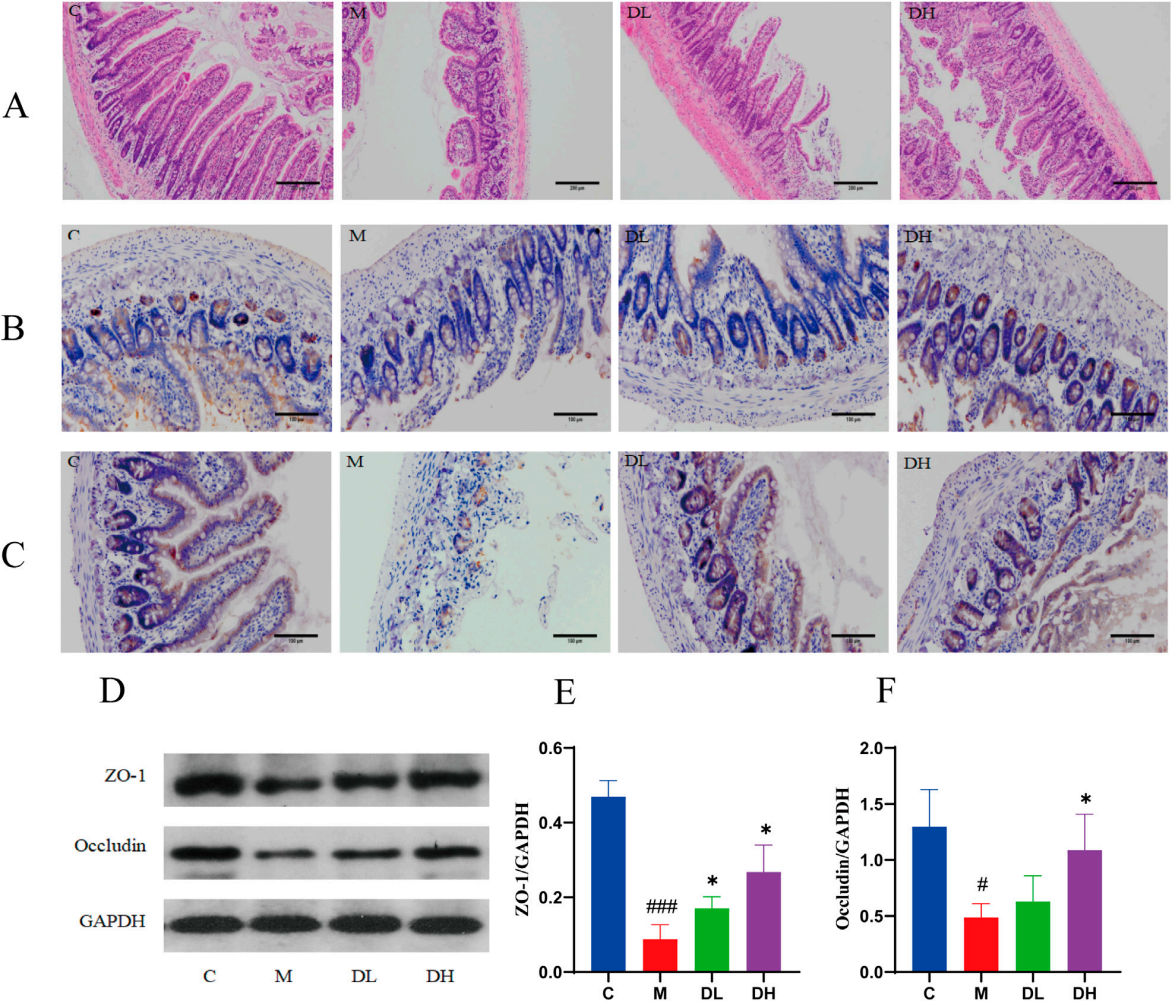
The total flavonoids of *Dracocephalum moldavica L.* improve intestinal barrier function in MAFLD rats. **(A)** HE staining of ileum (n = 4). **(B)** Ileal Z O-1 immunohistochemical staining (n = 3). **(C)** Ileal Occludin immunohistochemic al staining (n = 3). **(D–F)** ZO-1, Occludin protein expression levels in ileal tissue s (n = 3). Data are shown as mean values ± *S*. Compared with the C group,^#^ *P* < 0.05,^###^ *P* < 0.001, compared with the M group,^*^ *P* < 0.05,^**^ *P* < 0.01,^***^ *P* < 0.001.

Pathological analysis was performed by HE staining of rats ileum to evaluate the effect of the total flavonoids of *Dracocephalum moldavica L.* on the intestinal barrier of rats after intervention. The results of HE staining showed (Figure 6A) that the ileal villi of rats in the M group were impaired in detachment and the intestinal glands were sparsely arranged with a higher inflammatory cell infiltration compared to those in the C group. After the treatment with the total flavonoids of *Dracocephalum moldavica L.*, the structure of ileal villi was improved and the infiltration of inflammatory cell infiltration was reduced. The above results indicated that the total flavonoids of *Dracocephalum moldavica L.* inhibited intestinal epithelial necrosis, improved the structure of the intestinal villi, and effectively reduced the inflammatory cell infiltration in the intestinal mucosa of rats.

The tight junction proteins ZO-1 and Occludin play an important role in the regulation of intestinal permeability, and when this structure is disrupted, it leads to the translocation of LPS to the liver and blood system, causing liver inflammation and injury (Bein et al., 2017a). Therefore, in this experiment, we used immunohistochemistry to determine the expression of mucosal tight junction proteins ZO-1 and Occludin in the ileal tissues of rats in each group and used Western blotting to verify this result. The results of immunohistochemistry and Western blotting showed (Figures 6B–F) that the levels of ileal ZO-1 and Occludin proteins were decreased in the M group compared with the C group (*P* < 0.05 or *P* < 0.001); and the levels of ZO-1 and Occludin proteins were increased after the intervention of the total flavonoids of *Dracocephalum moldavica L.* compared with the M group (*P* < 0.05). These results suggest that the total flavonoids of *Dracocephalum moldavica L.* can effectively improve the integrity of the intestinal barrier after the intervention of *Dracocephalum moldavica L.*

### 3.7 The total flavonoids of *Dracocephalum moldavica L.* ameliorate liver inflam mation in MAFLD rats via TLR4/MyD88/NF-κB signalling pathway

The LPS plays a mediating role in diseases of the entero-liver axis diseases. The LPS released from the gut microbiota is absorbed via the intestinal myocoagulant blood circulation, and reaches the liver by the portal vein. When the LPS content exceeds the liver’s ability to process, the organism will form enterogenous endotoxemia (Barchetta et al., 2023). The intracellular Toll-like receptor (TLR) plays a key role in the hepatic processing of LPS. TLR4 recognizes LPS released by bacteria and initiates signaling pathways that result in the release of a multitude of inflammatory cytokines and the initiation of an immune-inflammatory response, which mediates hepatic inflammation.TLR4 can mediate hepatic inflammation by initiating the activation of the myeloid differentiation factor 88 (MyD 88). TLR4 can exacerbate the development of MAFLD by activating MyD88-dependent activation of NF-κB, which ultimately leads to the release of inflammatory factors, such as TNF-α, IL-6, and IL-1β (Ding et al., 2023; Khanmohammadi and Kuchay, 2022a; Sharifnia et al., 2015).

**FIGURE 7:**
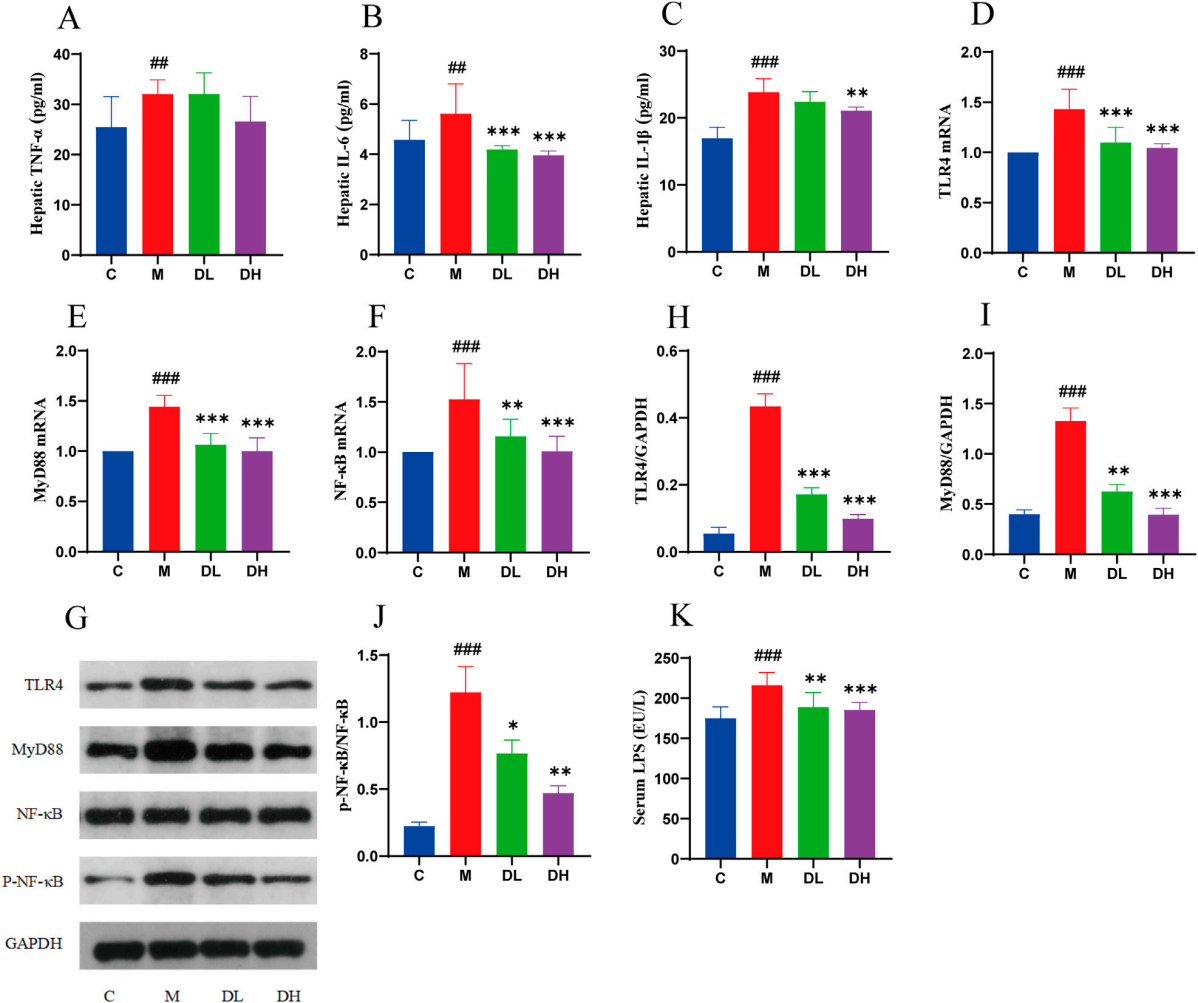
The total flavonoids of *Dracocephalum moldavica L.* ameliorate liver inflammation in MAFLD rats through TLR4/MyD88/NF- κB signalling pathway. **(A–C)** Hepatic TNF-α, IL-6, IL-1β levels(n = 6). **(D–F)** TLR4, MyD88, NF- κB mRNA livers(n = 6). **(G–J)** Expression levels of TLR4, MyD88, and p-NF- κB/NF- κB proteins in the liver(n = 3). **(K)** Serum LPS level (n = 8). Data are shown as mean values ± *S*. Compared with the C group,^##^ *P* < 0.01,^###^ *P* < 0.001, compared with the M group,^*^ *P* < 0.05,^**^ *P* < 0.01,^***^ *P* < 0.001.

Therefore, the experiment detected the level of LPS in the blood, and the expression content of TLR4/MyD88/NF-κB in the liver using RT-qPCR and Western blotting(Figures 7A–J). The LPS results demonstrated that the serum level of the LPS in rats in the M group was significantly increased compared with the C group. Following the administration of the total flavonoids of *Dracocephalum moldavica L.*, the level of the LPS in rats was significantly elevated. The RT-qPCR results showed that the expression of TLR4, MyD88 and NF-κB in the liver tissue of rats in the M group was significantly higher compared with the C group, while the total flavonoids of *Dracocephalum moldavica L.* significantly reduced the expression of TLR4, MyD88 and NF-κB in the liver tissue of rats with MAFLD after the intervention of the M group. Western blotting results showed that the expression of TLR4, MyD88, and p-NF-κB was significantly lower in the liver tissue of rats in the M group compared with the C group. The expression of TLR4, MyD88, p-NF-κB protein in the liver tissue of the M group rats was significantly reduced compared with that of the C group. The intervention of the total flavonoids of *Dracocephalum moldavica L.* effectively downregulated the expression of TLR4, MyD88, p-NF-κB protein in the liver tissue of MAFLD rats. These results demonstrated that the total flavonoids of *Dracocephalum moldavica L.* may improve liver inflammation by regulating TLR4/MyD88/NF-κB signaling pathway.

## 4 Discussion

The expansion of social and economic development, coupled with an uptick in living standards, has led to a surge in the prevalence of MAFLD, which has now emerged as one of the most prevalent liver diseases and a significant public health concern, affecting the health of the global population (Vitale et al., 2023). The pathogenesis of MAFLD is complex, and the main interventions at currently available are a reasonable diet and appropriate exercise. There is currently no approved therapeutic drug for MAFLD, and there is an urgent need to develop new effective drugs. Traditional Chinese medicines and natural products show great potential due to their multi-level, multi-component and multi-target characteristics. Chinese herbs and natural products show great potential due to their multi-level, multi-component and multi-target characteristics (Wong, 2018; Salvoza et al., 2022). As a natural product, the total flavonoids of *Dracocephalum moldavica L.* have previously been found to have anti-inflammatory, antioxidant and insulin resistance-relieving effects, and to regulate the gut microbiota, thus playing a role in preventing and controlling colitis. Consequently, the present study investigated the interventional effects of the total flavonoids of *Dracocephalum moldavica L.* on MAFLD induced by HFD.

The results indicated that the weight and liver weight of rats in the M group increased, accompanied by elevated serum levels of AST, ALT, TC, TG, LDL-C and inflammatory factors TNF-α, IL-6, IL-1β levels increased, HDL-C levels decreased. Furthermore, HE and Oil Red O staining revealed that the liver of rats in the M group was deposited with lipids. These findings collectively indicate that HFD successfully induced the establishment of MAFLD models in rats. The administration of the total flavonoids of *Dracocephalum moldavica L.* significantly attenuated the HFD-induced increase in body weight and liver weight, and reduced the hepatic lipid deposition and the expression of inflammatory factors. These findings suggest that the total flavonoids of *Dracocephalum moldavica L.* may be effective in mitigating development of MAFLD induced by HFD. The results indicate that the total flavonoids of Dracocephalum moldavica L. may have potential as a treatment for MAFLD.

The development of MAFLD is closely related to disorders of the gut microbiota. The liver and intestine are connected through the portal vein, which renders the liver is susceptible to bacterial translocation (Stachowska et al., 2020). 16Sr RNA gene sequencing was used to examine the composition and structure of the gut microbiota in various groups of rats. Alpha and Beta diversity results demonstrated that the total flavonoids of *Dracocephalum moldavica L.* could prevent MAFLD by increasing the abundance and diversity of the gut microbiota. At the phylum level, the ratio of Firmicutes to Bacteroidetes was found to be associated with metabolic diseases such as obesity. At the genus level, there are significant differences between Akkermansia, Romboutsia, Allobaculum, Muribaculum, *Lactobacillus*, Lachnoclostridium, and Blautia. Romboutsia is strongly associated with inflammatory responses, and some studies have demonstrated that Romboutsia is a marker of MAFLD to NASH transition (Zhuge et al., 2022). Allobaculum alleviates MAFLD by producing a large amount of short-chain fatty acids to activate AMPK/Nrf2 signalling (Peng et al., 2024). It has been suggested that by increasing the relative abundance of Lachnoclostridium spp. may be an effective way to reduce the risk of MAFLD (Dai et al., 2023b). The aforementioned findings indicate that the total flavonoids of *Dracocephalum moldavica L.* exhibit a protective effect against MAFLD, as evidenced by their ability to reduce the population of harmful bacteria and to enhance the growth of beneficial bacteria.

The “two-hit” hypothesis suggests that MAFLD is not solely a hepatic manifestation of metabolic syndrome; rather, it is a multifaceted condition that affects a range of extrahepatic organs and tissues, including the intestines and adipose tissues. These organs and tissues are closely related to the development of MAFLD, and the leakage of intestinal LPS may be an important cause of persistent hepatic inflammatory injury and the development of MAFLD. (Fang et al., 2018; Bahitham et al., 2024; Song and Zhang, 2022). The intestinal mucosal barrier is capable of resisting intestinal bacterial translocation and reducing LPS leakage, and is regarded as the “first line of defence” against enteric pathogens. The primary layer of the barrier is the intestinal mechanical barrier, and the tight junction is the fundamental structure of the intestinal mechanical barrier (Safari and Gérard, 2019; Bein et al., 2017b). Tight junctions are composed of Occludin, a structural protein, and ZO-1, a linker protein, which are tightly connected to the intestinal epithelial cells in the form of a hoop (Bocsik et al., 2016; Tripathi et al., 2018). It has been postulated that hypertriglyceridemia and high plasma LPS associated with HFD may be due to decreased expression of intestinal epithelial proteins such as Occludin and ZO-1 secondary to increased intestinal permeability (Wang et al., 2021; Dembek et al., 2017; Xu et al., 2007). In this experiment, the expression and distribution of tight junction proteins Occludin and ZO-1 in the intestines of rats in each group were measured by Western blotting and immunohistochemical staining in order to assess the intestinal mucosal barrier function of rats in each group. Upon microscopic examination, it was observed that Occludin and ZO-1 were distributed in the ileal tissue in brown color on the surface of the intestinal mucosa and in the crypts of the intestinal mucosa, and were arranged in a line around the cells of the mucosal layer. The results of Western blotting demonstrated that the expression of Occludin and ZO-1 in the ileal tissue of the rats in the M group was reduced in comparison with that of the rats in the C group. Following the intervention of the total flavonoids of *Dracocephalum moldavica L.,* the expression of Occludin and ZO-1 in the ileal tissue of rats in the DH group was significantly higher than that of rats in the M group. The above results indicated that the total flavonoids of *Dracocephalum moldavica L.* could restore the integrity of the intestinal mucosa damaged by HFD through the upregulation of the expression of tight junction proteins.

When the intestinal mucosal barrier is disrupted and the intestinal mucosal permeability is increased, the LPS will pass through the intestinal mucosal barrier into the bloodstream and then into the liver, where it will activate hepatocytes through the pathogen-associated molecular patterns of the body’s natural immune system and the molecular patterns. The injury will result in the initiation of an inflammatory response by kupffer cells and the intracellular Toll-like receptor-TLR, in which the TLR4 is able to recognize the LPS released by the bacteria and carry out the corresponding signaling. There are two downstream pathways of TLR4, mainly MyD88-dependent and MyD-independent (Ren et al., 2024b; Khanmohammadi and Kuchay, 2022b; Shen et al., 2020; Wang et al., 2020). It has been demonstrated that knockdown of the MyD88 gene can effectively prevent steatohepatitis caused by choline deficiency in mice, thereby indicating that MyD88 plays an important role in the development of MAFLD. Furthermore, the MyD88-dependent pathway can lead to the activation of NF-κB, which ultimately leads to the release of inflammatory factors TNF-α, IL-6, and IL-1β, and thus exacerbates the development of MAFLD (Yang et al., 2022; Wu et al., 2020; Lei et al., 2023). Both Western blotting and qPCR results demonstrated that the relative expression of TLR4, MyD88, and p-NF-κB in the liver tissues of rats in the M group was significantly increased compared with the C group (*P* < 0.01 or *P* < 0.001), and the total flavonoids of *Dracocephalum moldavica L.* could effectively inhibit the expression of TLR4, MyD88, and p-NF-κB in liver tissues (*P* < 0.05 or *P* < 0.01 or *P* < 0.001). These findings indicated that the total flavonoids of *Dracocephalum moldavica L.* could effectively suppress liver inflammation and improve MAFLD by inhibiting the LPS/TLR4/MyD88/NF-κB pathway.

However, this study also has some limitations. While there is relevant literature supporting that the total of *Dracocephalum L.* have advantages such as multi-pathway, multi-target, and multi-effect pharmacological actions, there are also many drug-related scientific issues, such as whether batch-to-batch quality variability significantly impacts the stability of efficacy and safety of the total flavonoids of *Dracocephalum moldavica L.*, and whether there is a PK-DDI risk if the total flavonoids of *Dracocephalum moldavica L.* are used in combination with other drugs in clinical settings. In the future, we will validate these issues. Additionally, this study lacks research on the TLR4/MyD88/NF-κB signaling pathway at the cellular level, and we will further improve this aspect in our future work.

In conclusion, the total flavonoids of *Dracocephalum moldavica L.* have the potential to restore the lipid metabolism level of MAFLD rats by inhibiting HFD-induced hepatic steatosis, hepatic inflammation and hepatic damage. Furthermore, the total flavonoids of *Dracocephalum moldavica L.* restored the barrier function of the intestinal mucosa and hindered the translocation of intestinal bacteria and LPS, which in turn inhibited the expression of the hepatic TLR4/MyD88/NF-κB pathway and ameliorated the development of MAFLD.

## Data Availability

The original contributions presented in the study are included in the article/Supplementary Material, further inquiries can be directed to the corresponding author.
