# Supplementary material for: Effect of the total flavonoids of Dracocephalum moldavica L. on metabolic associated fatty liver disease in rats
Source: Front Pharmacol. 2025 May 15;16:1549515. doi: 10.3389/fphar.2025.1549515 (PMC12119540; doi:10.3389/fphar.2025.1549515)
Supplement: Supplementary file 1 [file DataSheet1.pdf]

Supplementary Material 1

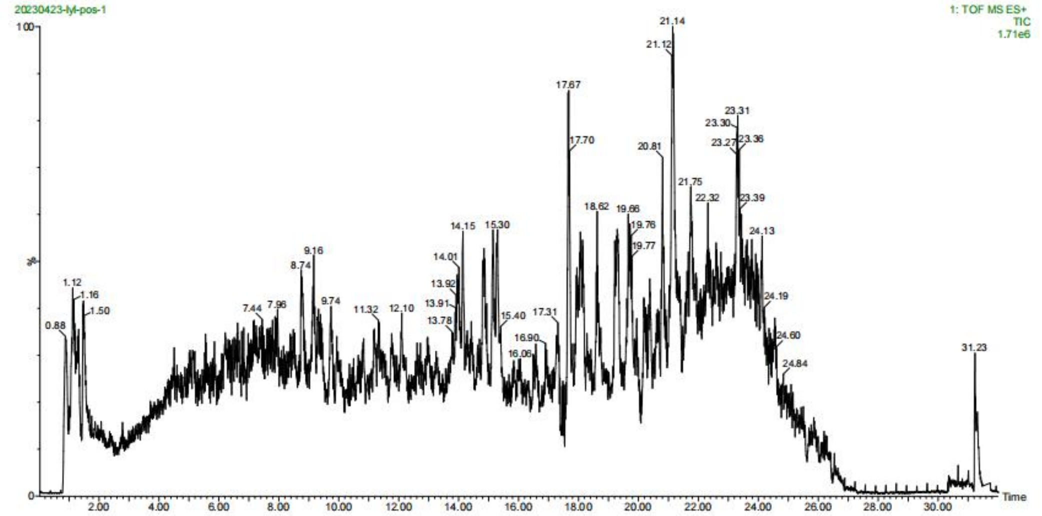

Positive ion spectrum

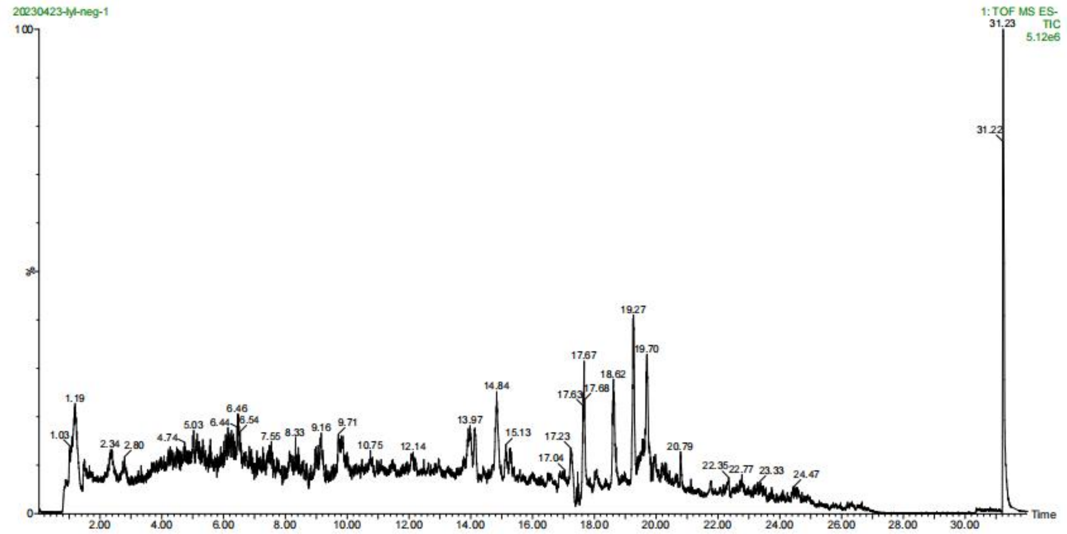

Negative ion spectrum

Number	Retention time/min	Molecular formula	Mode	Pseudo - molecular ion	The MS^2 spectrum from the MassBank database	Chemical compound
1	2.23	C18H16O8	$[M-H]^-$	359.0981	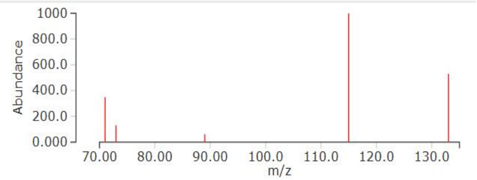	Rosmarinic acid
2	5.98	C22H22O10	$[M+H]^+$	447.0946	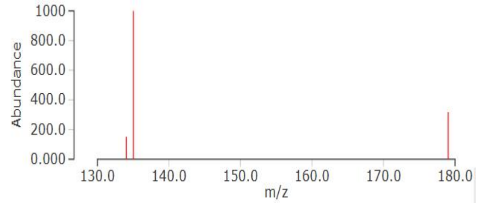	Tilianin
3	6.23	C21H20O10	$[M+H]^+$	433.1140	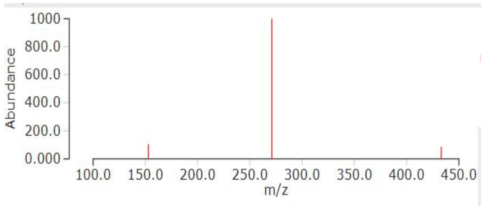	Cosmosiin

4	7.55	C15H10O6	$[M+H]^{+}$<br>$[M-H]^{-}$	287.0569<br>285.0398	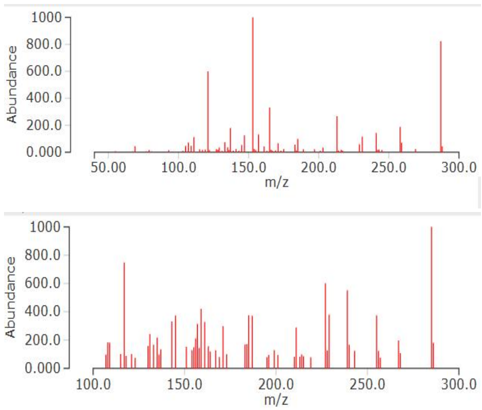	Kaempferol
5	8.51	C15H10O5	$[M-H]^{-}$<br>$[M+H]^{+}$	269.0449<br>271.0605	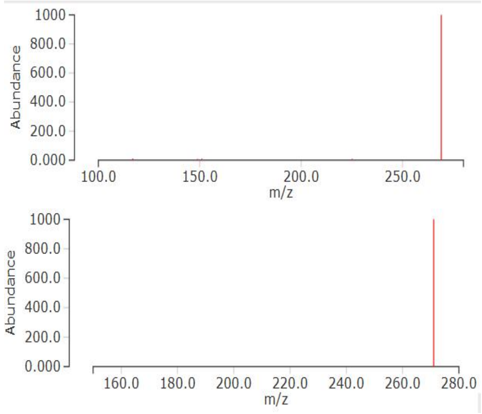	Apigenin

6	8.69	C16H12O6	[M+H]$^{+}$<br>[M-H]$^{-}$	301.0710<br>299.0551	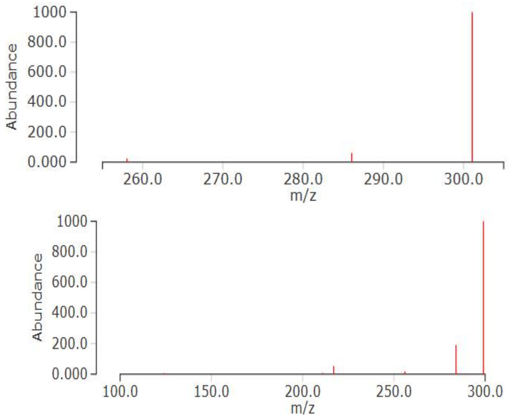	chrysoeriol
7	9.60	C7H6O5	[M-H]$^{-}$	169.0868	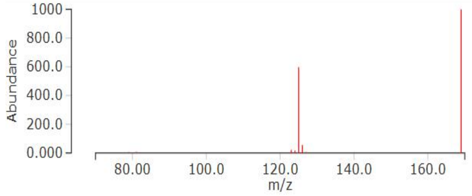	Gallic acid
8	10.66	C16H12O5	[M+H]$^{+}$<br>[M-H]$^{-}$	285.0762<br>283.0607	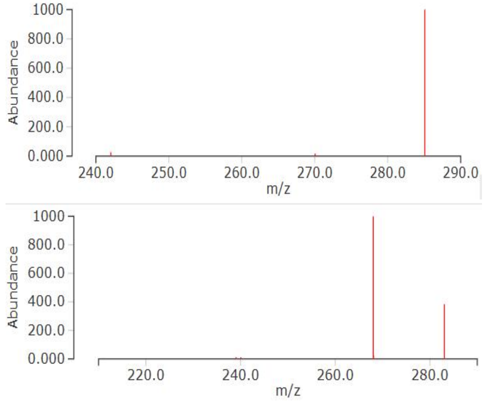	Acacetin

9	10.76	C15H14O6	$[M+H]^+$	291.1955	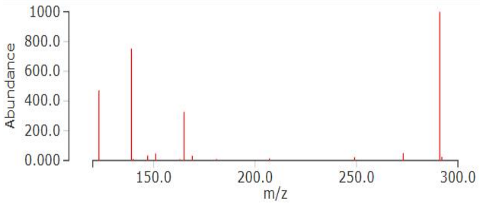	Epicatechin
10	11.28	C15H10O3	$[M-H]^-$	237.1488	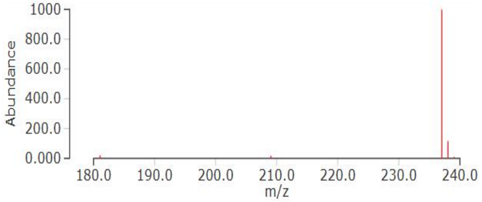	3-hydroxyflavone
11	12.19	C12H12O2	$[M+H]^+$	165.0909	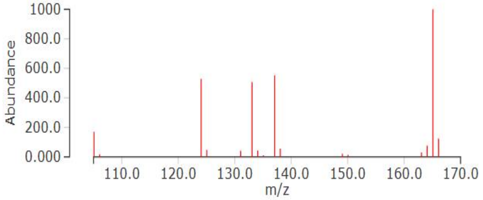	Eugenol
12	12.91	C29H50O	$[M-H]^-$	413.2543	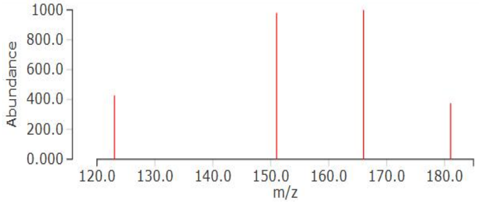	β-Sitosterol
13	13.38	C19H18O8	$[M-H]^-$	373.2017	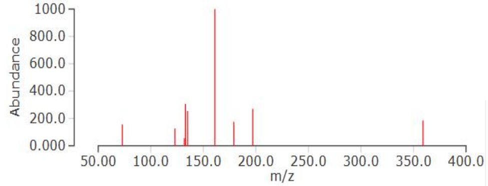	Methyl rosmarinate

14	13.74	$C_{16}H_{12}O_7$	$[M-H]^-$	315.2540	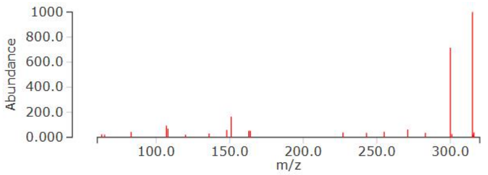	Isorhamnetin
15	18.84	$C_{27}H_{32}O_{14}$	$[M-H]^-$	579.2834	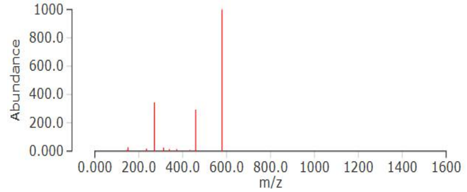	Naringin
16	18.62	$C_{18}H_{32}O_2$	$[M+H]^+$<br>$[M-H]^-$	281.2479<br>279.2327	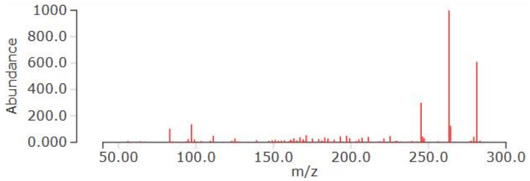<br>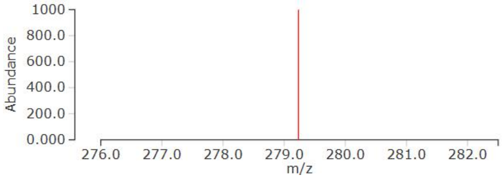	linoleic acid
